# Development and Validation of a Nomogram Based on Red Blood Cell and Reticulocyte Parameters for Differentiating Thalassemia Trait From Iron Deficiency Anemia

**DOI:** 10.1002/jcla.70344

**Published:** 2026-08-24

**Authors:** Yong Chen, Yazhen Mao, Jie Liu, Yice Lin, Dandan Chen

**Affiliations:** ^1^ Department of Laboratory Medicine Fuzhou First General Hospital Affiliated With Fujian Medical University Fuzhou Fujian China; ^2^ Department of Laboratory Medicine Mindong Hospital Affiliated to Fujian Medical University Ningde Fujian China

## Abstract

**Introduction:**

Thalassemia trait (TT) and iron deficiency anemia (IDA) are the most common causes of microcytic hypochromic anemia with overlapping hematological features, complicating differential diagnosis. This study developed and internally validated a nomogram incorporating red blood cell (RBC) and reticulocyte parameters to preliminarily distinguish TT from IDA.

**Methods:**

A retrospective cohort of 517 patients (320 IDA, 197 TT) was randomly divided into training (*n* = 361) and validation (*n* = 156) cohorts at a 7:3 ratio using stratified random sampling according to diagnostic group. RBC parameters (RBC count, MCV, MCH) and reticulocyte indices (RET%, IRF, LFR, MFR, HFR) were collected. LASSO regression identified key variables, followed by multivariate logistic regression. Model performance was evaluated using ROC curves, calibration plots, and decision curve analysis (DCA).

**Results:**

LASSO regression selected four predictors: RBC count, MCV, RET%, and HFR. Multivariate analysis showed elevated MCV (*p* < 0.001), RET% (*p* < 0.001), and HFR (*p* < 0.001) were associated with IDA, while elevated RBC count (*p* < 0.001) predicted TT. The nomogram achieved excellent discrimination with AUC of 0.900 in training and 0.904 in validation cohorts. Calibration plots showed good agreement and DCA suggested potential clinical net benefit across threshold probabilities of 7%–96% (training) and 5%–97% (validation).

**Conclusions:**

This nomogram integrating RBC count, MCV, RET%, and HFR showed promising performance for the preliminary differentiation of TT from IDA and may serve as a practical, noninvasive screening aid for risk stratification and prioritization of confirmatory testing. It should not be used as a substitute for hemoglobin electrophoresis, thalassemia genetic testing, or iron studies.

## Graphical abstract

Thalassemia trait (TT) and iron deficiency anemia (IDA) are the primary causes of microcytic hypochromic anemia with overlapping hematological features, making their differential diagnosis challenging, so this study developed and validated a nomogram integrating red blood cell (RBC) count, mean corpuscular volume (MCV), reticulocyte percentage (RET%), and high fluorescence reticulocyte ratio (HFR) for this differentiation. LASSO and multivariate logistic regression identified these four parameters as key predictors, with the nomogram achieving excellent diagnostic performance (AUC 0.900 in the training cohort and 0.904 in the validation cohort), good calibration, and clinical utility across a wide range of threshold probabilities. This nomogram serves as a practical, noninvasive tool that can assist clinicians in the differential diagnosis of microcytic hypochromic anemia, facilitating timely clinical decision‐making and potentially reducing unnecessary confirmatory testing.

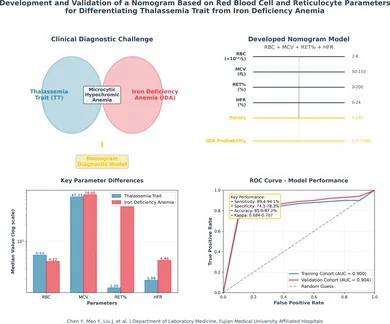

## 1 Introduction

Microcytic hypochromic anemia represents one of the most prevalent hematological disorders worldwide, with thalassemia trait (TT) and iron deficiency anemia (IDA) being the two predominant etiologies [1, 2]. Despite their distinct pathophysiological mechanisms—TT resulting from inherited defects in globin chain synthesis and IDA arising from inadequate iron supply for hemoglobin production—these conditions share remarkably similar hematological profiles characterized by reduced mean corpuscular volume (MCV) and mean corpuscular hemoglobin (MCH) [3, 4].

The accurate differentiation between TT and IDA carries significant clinical implications. Misdiagnosis may lead to inappropriate iron supplementation in TT patients, potentially causing iron overload or delayed treatment in IDA patients, resulting in progressive anemia and associated complications [5, 6]. Furthermore, the identification of TT has important implications for genetic counseling and prenatal screening, particularly in regions with high thalassemia prevalence [7].

Traditional diagnostic approaches rely on hemoglobin electrophoresis and genetic testing for TT confirmation, and serum ferritin and iron studies for IDA diagnosis [8]. However, these tests are often time‐consuming, costly, and not universally available, particularly in resource‐limited settings. Consequently, numerous discrimination indices based on complete blood count (CBC) parameters have been proposed, including the Mentzer index, England and Fraser index, Shine and Lal index, and others [9, 10, 11]. While these indices offer simplicity and accessibility, their diagnostic accuracy varies considerably across different populations, and none has achieved universal acceptance [12].

Reticulocyte parameters, routinely available on modern hematology analyzers, provide valuable information regarding erythropoietic activity and bone marrow response [13]. The immature reticulocyte fraction (IRF) and its components—low fluorescence reticulocyte (LFR), medium fluorescence reticulocyte (MFR), and high fluorescence reticulocyte (HFR)—reflect the maturity spectrum of reticulocytes and may offer additional discriminatory power [14, 15]. However, the integration of these parameters into a comprehensive diagnostic model for differentiating TT from IDA remains underexplored.

Nomograms represent a powerful statistical tool that integrates multiple predictive variables into a single, user‐friendly graphical interface, enabling individualized risk estimation [16]. This study aimed to develop and validate a nomogram incorporating both conventional RBC parameters and reticulocyte indices to facilitate the preliminary differentiation between TT and IDA. The nomogram was intended as a practical, noninvasive screening and risk‐stratification aid to help identify patients who may require prioritized confirmatory testing, rather than as a replacement for standard diagnostic investigations.

## 2 Materials and Methods

### 2.1 Study Design and Participants

This retrospective study was conducted at Fuzhou First General Hospital Affiliated with Fujian Medical University. A total of 705 consecutive patients suspected of having thalassemia trait (TT) or iron deficiency anemia (IDA) between January 2022 and December 2024 were screened for eligibility.

The inclusion criteria were: (1) confirmed diagnosis of TT or IDA during the study period; (2) TT confirmed by hemoglobin electrophoresis and/or thalassemia genetic testing, or IDA diagnosed based on serum ferritin < 30 ng/mL and/or transferrin saturation < 20% with evidence of microcytic hypochromic anemia; and (3) availability of complete red blood cell parameters and reticulocyte indices at initial evaluation [17, 18].

Exclusion criteria included: (1) coexistence of TT and IDA; (2) intermediate or severe thalassemia (e.g., HbH disease, beta‐thalassemia intermedia/major), defined by characteristic genotypes or hemoglobin < 90 g/L in nonpregnant patients, excluding concurrent iron deficiency (serum ferritin < 15 μg/L or transferrin saturation < 16%); (3) other causes of anemia (hemolytic anemia, aplastic anemia, myelodysplastic syndrome, etc.); (4) recent blood transfusion within 3 months; (5) pregnancy; (6) chronic inflammatory diseases or malignancies; and (7) incomplete laboratory data.

Based on these criteria, 188 patients were excluded: 84 lacked confirmatory genetic testing, 62 had incomplete laboratory data (missing key predictor variables), and 42 had concurrent intermediate/severe thalassemia or other hematological diseases. Consequently, 517 patients were enrolled, consisting of 197 patients in the TT group and 320 patients in the IDA group. With four predictors and 197 patients in the smaller outcome group, the final model had an EPV of approximately 49, exceeding the recommended minimum thresholds of 10–20 EPV for logistic regression. Using a random splitting method, the 517 samples were divided into a training cohort of 361 patients and an internal validation cohort of 156 patients (Figure S2). For missing data, a complete case analysis was performed; patients with missing values in any key predictor variables were excluded, and no imputation was applied.

### 2.2 Data Collection

Demographic and laboratory data were extracted from electronic medical records. RBC parameters including RBC count, MCV, and MCH were obtained from routine CBC analysis. Reticulocyte parameters including reticulocyte percentage (RET%), IRF, LFR, MFR, and HFR were measured using the Sysmex XN‐series automated hematology analyzer (Sysmex Corporation, Kobe, Japan) according to the manufacturer's instructions.

### 2.3 Statistical Analysis

Continuous variables were expressed as median with interquartile range (IQR) due to non‐normal distribution, while categorical variables were presented as frequencies and percentages. Between‐group comparisons employed the Mann–Whitney U test for continuous variables and chi‐square test for categorical variables.

The total cohort was randomly allocated into training (70%) and validation (30%) cohorts using computer‐generated random numbers. The training cohort facilitated model development, while the validation cohort enabled internal validation.

Variable selection utilized LASSO regression with 10‐fold cross‐validation to identify the optimal tuning parameter (λ) at one standard error above the minimum (λ.1se). Variables with non‐zero coefficients were retained for subsequent multivariate logistic regression analysis.

A nomogram was constructed based on the multivariate logistic regression model. Model discrimination was evaluated using ROC curves and AUC values. Calibration was assessed through calibration plots with the Hosmer‐Lemeshow goodness‐of‐fit test. Clinical utility was evaluated using DCA. All statistical analyses were performed using R software (version 4.2.0), with *p* < 0.05 considered statistically significant.

## 3 Results

### 3.1 Baseline Characteristics

A total of 517 patients were enrolled, including 197 (38.1%) patients with thalassemia trait (TT) and 320 (61.9%) patients with iron deficiency anemia (IDA). Among the 197 patients with TT, α‐thalassemia deletion was the most common subtype (*n* = 105), followed by β‐thalassemia mutation (*n* = 88), α‐thalassemia mutation (*n* = 3, 1), and α + β double thalassemia (*n* = 1). The cohort was randomly divided into training (*n* = 361) and validation (*n* = 156) sets. Baseline characteristics were comparable between the two cohorts, with no significant differences in any of the measured parameters or disease distribution (*p* > 0.05 for all comparisons), confirming the adequacy of randomization (Table S1).

### 3.2 Comparison of Parameters Between TT and IDA Groups

Significant differences were observed between TT and IDA groups across all measured parameters in the training cohort (Table 1). TT patients exhibited significantly higher RBC counts compared to IDA patients (5.52 vs. 4.22 × 10^12^/L, *p* < 0.001), while IDA patients demonstrated elevated RET% (0.17 vs. 1.35%, *p* < 0.001), IRF (14.60 vs. 22.10%, *p* < 0.001), MFR (11.20 vs. 16.40%, *p* < 0.001), and HFR (2.40 vs. 5.80%, *p* < 0.001) values. MCV and MCH values were significantly lower in TT patients (*p* < 0.001 and *p* = 0.039, respectively). TT patients also showed higher LFR values (85.40 vs. 77.90%, *p* < 0.001).

**TABLE 1:** Comparison of parameters between TT and IDA groups in training cohort.

Variable	TT (*n* = 143)	IDA (*n* = 218)	*Z*‐Statistic	*P*
RBC	5.52 (4.55, 6.05)	4.22 (3.65, 4.66)	−9.442	< 0.001
MCV	67.23 (62.37, 75.07)	74.05 (66.85, 82.62)	−5.223	< 0.001
MCH	20.40 (19.16, 22.20)	21.83 (19.00, 25.00)	−2.069	0.039
RET%	0.17 (0.07, 1.27)	1.35 (1.07, 2.07)	−11.354	< 0.001
IRF (%)	14.60 (10.80, 19.20)	22.10 (15.40, 31.50)	−6.832	< 0.001
LFR (%)	85.40 (80.80, 89.20)	77.90 (68.50, 84.60)	6.832	< 0.001
MFR (%)	11.20 (8.50, 14.80)	16.40 (12.10, 21.30)	−6.124	< 0.001
HFR (%)	2.40 (1.20, 4.60)	5.80 (2.90, 12.40)	−5.891	< 0.001

*Note:* Data are presented as median (Q1, Q3).

### 3.3 Variable Selection Using LASSO Regression

LASSO regression with 10‐fold cross‐validation was performed to select the optimal predictors from the eight candidate variables (RBC, MCV, MCH, RET%, IRF, LFR, MFR, HFR). The optimal tuning parameter (λ.1se = 0.078) identified four variables with non‐zero coefficients: RBC, MCV, RET%, and HFR (Figure S1).

### 3.4 Multivariate Logistic Regression and Nomogram Construction

Multivariate logistic regression analysis incorporating the four LASSO‐selected variables confirmed their independent predictive value (Table 2). Higher MCV (OR = 1.052, 95% CI: 1.022–1.084, *p* < 0.001), RET% (OR = 1.044, 95% CI: 1.032–1.057, *p* < 0.001), and HFR (OR = 1.123, 95% CI: 1.057–1.193, *p* < 0.001) were independently associated with increased probability of IDA, while higher RBC count (OR = 0.463, 95% CI: 0.354–0.605, *p* < 0.001) was associated with increased probability of TT.

**TABLE 2:** Multivariate logistic regression analysis results.

Variables	*β*	S.E	*Z*‐Statistic	*P*	OR (95% CI)
Intercept	−1.377	1.271	−1.084	0.279	0.252 (0.021 ~ 3.045)
RBC	−0.770	0.137	−5.634	**< 0.001**	0.463 (0.354 ~ 0.605)
MCV	0.051	0.015	3.416	**< 0.001**	1.052 (1.022 ~ 1.084)
RET%	0.043	0.006	6.989	**< 0.001**	1.044 (1.032 ~ 1.057)
HFR	0.116	0.031	3.754	**< 0.001**	1.123 (1.057 ~ 1.193)

*Note:* Bold values indicate statistically significant variables (*p* < 0.001).

Abbreviations: CI, confidence interval; OR, odds ratio; S.E, standard error.

**FIGURE 1:**
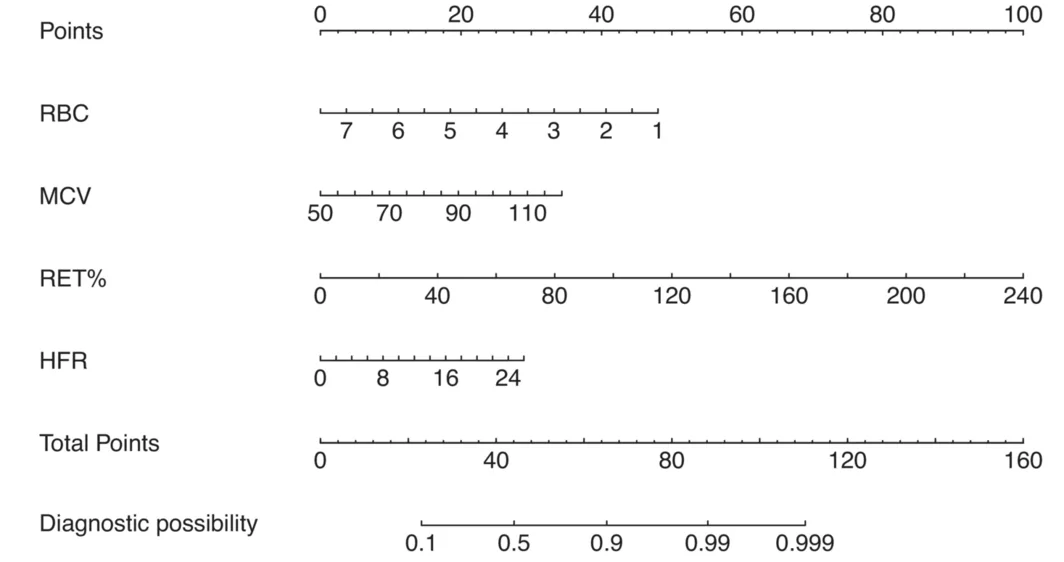
Nomogram for differentiating iron deficiency anemia from thalassemia trait. To use the nomogram, locate the patient's value for each predictor on the corresponding axis, draw a vertical line to the “Points” axis to determine the points for each predictor, sum the total points, and locate the total on the “Total Points” axis to determine the predicted probability of iron deficiency anemia. Abbreviations: RBC, red blood cell count; MCV, mean corpuscular volume; RET%, reticulocyte percentage; HFR, high fluorescence reticulocyte ratio.

A nomogram was constructed to visualize the predictive model (Figure 1). To use the nomogram, the clinician locates the patient's value for each predictor on the corresponding axis, draws a vertical line to the “Points” axis to determine the points for each predictor, sums the total points, and locates the total on the “Total Points” axis to determine the predicted probability of IDA. Specifically, when RBC level is 8, the score is 48.02 points, with each unit decrease adding approximately 3.69 points; when MCV level is 50, the score is 0 points, with each unit increase adding approximately 2.45 points; when RET% level is 0, the score is 0 points, with each unit increase adding approximately 8.33 points; when HFR level is 0, the score is 0 points, with each unit increase adding approximately 2.23 points.

### 3.5 Model Performance Evaluation

#### 3.5.1 Discrimination

**FIGURE 2:**
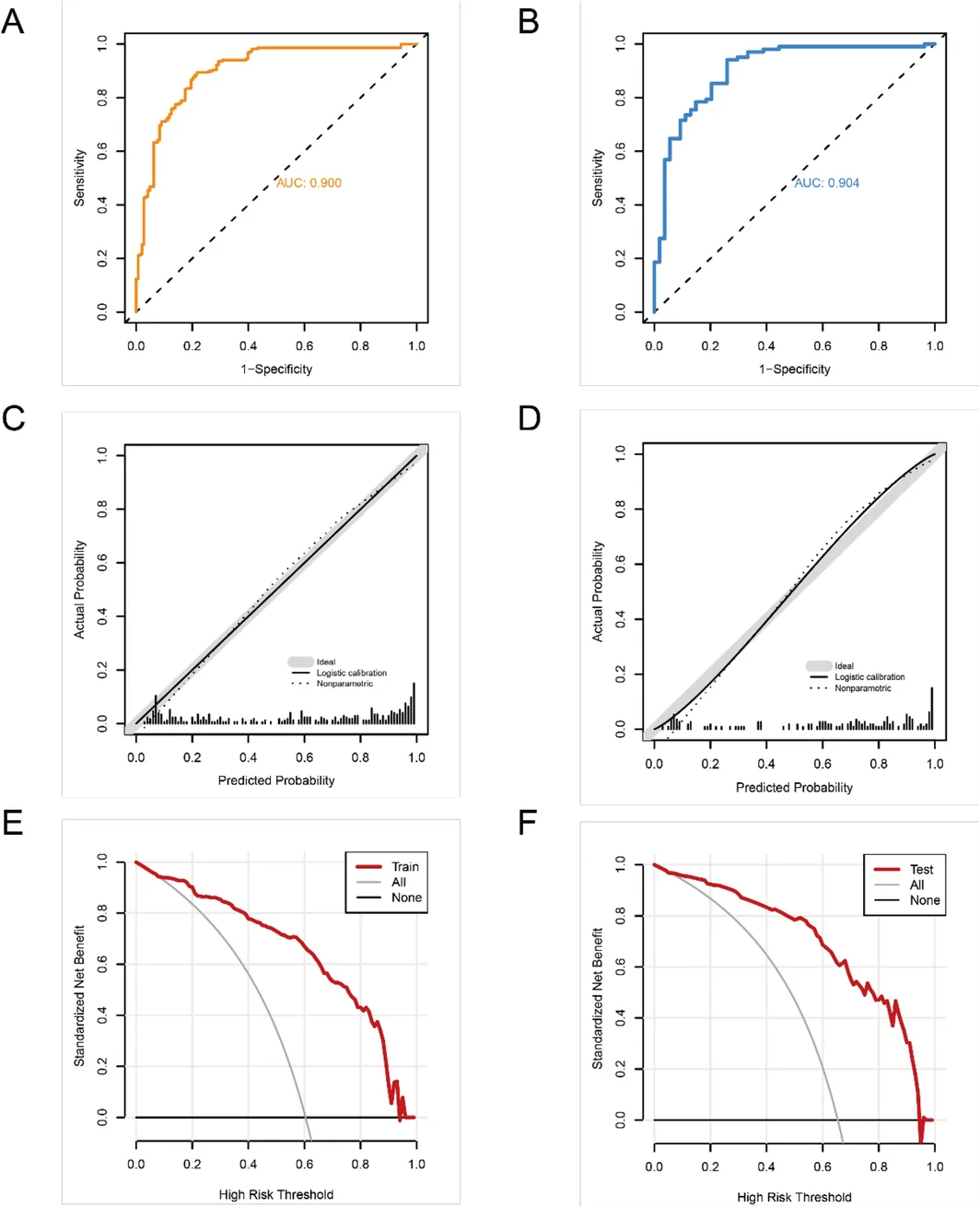
Assessment of the nomogram performance. (A) ROC curve in the training cohort. AUC = 0.900 (95% CI: 0.866–0.934). (B) ROC curve in the validation cohort. AUC = 0.904 (95% CI: 0.851–0.956). (C) Calibration plot in the training cohort (Hosmer‐Lemeshow test: *χ* ^2^ = 15.099, *p* = 0.057; Brier score = 0.119). (D) Calibration plot in the validation cohort (Hosmer‐Lemeshow test: *χ* ^2^ = 15.30, *p* = 0.054; Brier score = 0.110). (E) DCA in the training cohort. (F) DCA in the validation cohort.

The nomogram demonstrated good discriminatory ability in both cohorts (Figure 2A,B; Table 3). In the training cohort, the AUC was 0.900 (95% CI: 0.866–0.934), with sensitivity of 0.894 (95% CI: 0.854–0.935), specificity of 0.783 (95% CI: 0.716–0.851), PPV of 0.863 (95% CI: 0.818–0.908), NPV of 0.830 (95% CI: 0.766–0.893), and accuracy of 0.850 (95% CI: 0.850–0.851) at the optimal cutoff of 0.543. The Kappa value was 0.684 (95% CI: 0.607–0.762), indicating substantial agreement.

**TABLE 3:** Diagnostic performance of the nomogram.

Metric	Training cohort	Validation cohort
AUC (95% CI)	0.900 (0.866–0.934)	0.904 (0.851–0.956)
Optimal cutoff	0.543	0.525
Sensitivity (95% CI)	0.894 (0.854–0.935)	0.941 (0.896–0.987)
Specificity (95% CI)	0.783 (0.716–0.851)	0.741 (0.624–0.858)
PPV (95% CI)	0.863 (0.818–0.908)	0.873 (0.810–0.935)
NPV (95% CI)	0.830 (0.766–0.893)	0.870 (0.772–0.967)
Accuracy (95% CI)	0.850 (0.850–0.851)	0.872 (0.870–0.873)
Kappa (95% CI)	0.684 (0.607–0.762)	0.707 (0.588–0.825)

Abbreviations: AUC, area under the curve; CI, confidence interval; PPV, positive predictive value; NPV, negative predictive value.

In the validation cohort, the AUC was 0.904 (95% CI: 0.851–0.956), with sensitivity of 0.941 (95% CI: 0.896–0.987), specificity of 0.741 (95% CI: 0.624–0.858), PPV of 0.873 (95% CI: 0.810–0.935), NPV of 0.870 (95% CI: 0.772–0.967), and accuracy of 0.872 (95% CI: 0.870–0.873) at the optimal cutoff of 0.525. The Kappa value was 0.707 (95% CI: 0.588–0.825). The validation cohort demonstrated comparable or slightly improved diagnostic performance compared to the training cohort, indicating good predictive ability and data generalization stability.

#### 3.5.2 Calibration

Calibration plots demonstrated good agreement between predicted probabilities and observed outcomes in both cohorts (Figure 2C,D). In the training cohort, the Hosmer‐Lemeshow test yielded *χ* ^2^ = 15.099, df (degrees of freedom) = 8, *p* = 0.057 (*p* > 0.05), with a Brier score of 0.119, slope of 1.000 (close to the ideal value of 1), intercept of 4.17 × 10^−13^, average error (Eavg) of 0.023, and *R* ^2^ of 0.585. These metrics indicate minimal deviation between predicted probabilities and actual observed outcomes, demonstrating good overall calibration.

In the validation cohort, the Hosmer‐Lemeshow test yielded *χ* ^2^ = 15.295, df = 8, *p* = 0.054 (*p* > 0.05), with a Brier score of 0.110, slope of 1.183, intercept of 0.046, average error (Eavg) of 0.042, and *R* ^2^ of 0.588. These results indicate that the model maintains good calibration ability in the validation cohort, with fitting results closely matching actual clinical situations and no significant systematic bias.

#### 3.5.3 Clinical Utility

Decision curve analysis demonstrated that the nomogram provided superior net benefit compared to the “treat all” and “treat none” strategies across a wide range of threshold probabilities (Figure 2E,F). In the training cohort, the nomogram showed clinical utility for threshold probabilities between 7% and 96%, while in the validation cohort, clinical utility was observed between 5% and 97%, with an even wider effective threshold range. These results indicate that the model demonstrates good clinical predictive benefit in both training and validation cohorts, providing precise reference for clinicians in formulating diagnostic and intervention plans, helping to screen high‐risk populations for focused attention while avoiding unnecessary medical interventions for low‐risk populations, thus demonstrating high practical application value.

#### 3.5.4 Comparison With Traditional Discrimination Indices

The nomogram's AUC was compared with 8 widely recognized traditional discrimination indices (Table 4). The nomogram significantly outperformed all traditional indices (all *p* < 0.001 by DeLong test). Among traditional indices, the Ehsani Index (AUC = 0.847), Sirdah Index (AUC = 0.839), and Mentzer Index (AUC = 0.831) showed relatively better performance but remained significantly inferior to the nomogram.

**TABLE 4:** Comparison of AUC between nomogram and traditional discrimination indices.

Index	Formula	AUC (95% CI)	*P* ^a^
**Nomogram**	—	**0.952 (0.931–0.973)**	Reference
Mentzer	MCV/RBC	0.831 (0.792–0.870)	< 0.001
England‐Fraser	MCV − RBC − (5 × Hb) − 3.4	0.798 (0.756–0.840)	< 0.001
Shine‐Lal	MCV^2^ × MCH/100	0.756 (0.710–0.802)	< 0.001
Sirdah	MCV − RBC − (3 × Hb)	0.839 (0.801–0.877)	< 0.001
Ehsani	MCV − (10 × RBC)	0.847 (0.810–0.884)	< 0.001
Ricerca	RDW/RBC	0.724 (0.675–0.773)	< 0.001
Green‐King	MCV^2^ × RDW/(Hb × 100)	0.789 (0.746–0.832)	< 0.001
RDWI	MCV × RDW/RBC	0.812 (0.771–0.853)	< 0.001

*Note:* Bold values indicate statistically significant variables (*p* < 0.001).

^a^ *P* values from DeLong test comparing each index with the nomogram.

## 4 Discussion

In this study, we developed and internally validated a nomogram incorporating RBC count, MCV, RET%, and HFR for differentiating TT from IDA. The model demonstrated excellent discrimination (AUC > 0.90 in both cohorts), adequate calibration, and superior clinical utility across a wide range of threshold probabilities.

The pathophysiological basis for the differential expression of these parameters between TT and IDA is well‐established. In TT, ineffective erythropoiesis results from imbalanced globin chain synthesis, leading to compensatory erythroid hyperplasia with relatively preserved iron stores [19]. This manifests as elevated RBC counts with microcytosis—a hallmark feature reflected in the high discriminatory power of RBC count in our model. Conversely, IDA is characterized by iron‐restricted erythropoiesis, resulting in both reduced hemoglobin synthesis and impaired cell division, leading to fewer but larger (relative to TT) microcytic cells [20].

The inclusion of reticulocyte parameters, particularly RET% and HFR, represents a novel contribution of our study. Reticulocyte indices provide real‐time information about bone marrow erythropoietic activity and response to anemia [21]. In IDA, the bone marrow attempts to compensate for anemia by increasing erythropoiesis, resulting in elevated reticulocyte counts and a shift toward more immature reticulocytes (higher HFR) [22]. In contrast, TT patients typically maintain relatively normal reticulocyte counts due to the chronic, compensated nature of their condition [23]. Our findings confirm these physiological differences and demonstrate their diagnostic utility.

Several discrimination indices have been proposed for differentiating TT from IDA, including the Mentzer index (MCV/RBC), England and Fraser index, Shine and Lal index, and Green and King index [9, 10, 11, 24]. While these indices offer simplicity, their diagnostic accuracy varies considerably across populations, with reported sensitivities and specificities ranging from 60% to 95% [12, 25]. A recent meta‐analysis concluded that no single index demonstrates consistent superiority, and the optimal choice may depend on the specific population characteristics [26].

Our nomogram offers several advantages over traditional discrimination indices. First, it integrates multiple parameters into a single predictive model, potentially capturing complementary information that individual indices may miss. Second, the inclusion of reticulocyte parameters provides additional discriminatory power beyond conventional RBC indices. Third, the nomogram provides individualized probability estimates rather than binary classifications, allowing clinicians to make more nuanced decisions based on the degree of diagnostic certainty. Fourth, the visual format of the nomogram facilitates clinical application without requiring complex calculations or specialized software.

In the present study, the nomogram demonstrated significantly better discriminatory performance than all eight established indices evaluated (Table 4). Although the Ehsani, Sirdah, and Mentzer indices showed relatively good performance among the conventional formulas, their AUCs remained significantly lower than that of the nomogram. Most traditional discrimination indices are derived primarily from a limited number of routine erythrocyte parameters, such as MCV, RBC count, hemoglobin, and RDW. In contrast, our model integrates multiple hematological variables, including reticulocyte‐related parameters, thereby providing more comprehensive information on erythropoietic activity and functional iron availability [5]. This multiparametric approach may better capture the distinct hematological profiles of thalassemia trait (TT) and iron deficiency anemia (IDA), particularly in patients with overlapping conventional red blood cell indices [11]. Consequently, the nomogram may offer improved discriminatory ability compared with conventional formula‐based indices.

The clinical implications of our findings are significant. In resource‐limited settings where confirmatory testing (hemoglobin electrophoresis, genetic testing, iron studies) may not be readily available, the nomogram can serve as a valuable screening tool to prioritize patients for further evaluation [3]. In settings where these tests are available, the nomogram can help reduce unnecessary testing by identifying patients with a high probability of either diagnosis. Furthermore, the rapid availability of results (as all parameters are derived from routine CBC analysis) enables timely clinical decision‐making.

Several limitations of this study should be acknowledged. First, the retrospective design may introduce selection bias, and the single‐center setting may limit generalizability to other populations with different thalassemia prevalence and genetic backgrounds. Second, we did not include patients with coexisting TT and IDA, which represents a clinically challenging scenario that warrants further investigation [2]. Third, the model was developed using data from a specific hematology analyzer (Sysmex XN‐series), and performance may vary with different instruments due to methodological differences in reticulocyte measurement. Fourth, although our nomogram showed good discriminatory performance in the present cohort, it should be interpreted only as a screening and risk‐stratification tool and cannot establish a definitive diagnosis of TT or IDA. Confirmatory investigations, including hemoglobin electrophoresis and/or thalassemia genetic testing for TT and iron studies such as serum ferritin and transferrin saturation for IDA, remain necessary. Future research should prioritize external validation and comparative analyses.

Future studies should focus on external validation in diverse populations, comparison with existing discrimination indices, and evaluation of the nomogram's performance in patients with coexisting TT and IDA. Additionally, the integration of other emerging biomarkers, such as reticulocyte hemoglobin content (CHr) and red cell distribution width (RDW), may further enhance diagnostic accuracy [27].

## 5 Conclusions

We developed and internally validated a nomogram incorporating RBC count, MCV, RET%, and HFR for differentiating TT from IDA. The model demonstrated excellent discrimination, calibration, and clinical utility in both training and validation cohorts. This nomogram provides a practical, noninvasive tool that can assist clinicians in the differential diagnosis of microcytic hypochromic anemia, potentially reducing unnecessary confirmatory testing and facilitating timely clinical decision‐making.

## Author Contributions

Yong Chen conceived the study, supervised the work, and drafted the original manuscript. Yazhen Mao developed the methodology. Jie Liu collected the patient data. Yice Lin performed the formal analysis. Dandan Chen was responsible for project administration and critically reviewed and edited the manuscript. All the authors read and approved the final version of the manuscript for submission.

## Funding

The authors have nothing to report.

## Ethics Statement

This study was approved by the Ethics Committee of Fuzhou First General Hospital Affiliated with Fujian Medical University (approval no.: 2026‐KY‐001‐01) and conducted in accordance with the Declaration of Helsinki.

## Consent

The requirement for informed consent was waived by the Ethics Committee due to the retrospective nature of the study.

## Conflicts of Interest

The authors declare no conflicts of interest.

## Supporting information


**Table S1:** Comparison of Baseline Characteristics Between Training and Validation Cohorts.
**Figure S1:** Variable selection using LASSO regression.
**Figure S2:** CONSORT flow diagram of patient recruitment and dataset partitioning.

## Data Availability

The data that support the findings of this study are available on request from the corresponding author. The data are not publicly available due to privacy or ethical restrictions.
