# Supplementary material for: Development and Validation of a Nomogram Based on Red Blood Cell and Reticulocyte Parameters for Differentiating Thalassemia Trait From Iron Deficiency Anemia
Source: J Clin Lab Anal. 2026 Aug 24;40(19):e70344. doi: 10.1002/jcla.70344 (PMC13504082; doi:10.1002/jcla.70344)
Supplement: Supplementary file 1 [file JCLA-40-e70344-s001.docx]

**Table S1. Comparison of Baseline Characteristics Between Training and Validation Cohorts**

| **Variable** | | | **Total**  **(n = 517)** | **Training**  **(n = 361)** | **Validation**  **(n = 156)** | **Z-Statistic** | **P value** |
| --- | --- | --- | --- | --- | --- | --- | --- |
| **Age（Years）** | | | 29(23, 35) | 30 (24, 36) | 27 (21, 33) | -1.253 | 0.210 |
| **Gender,n(%)** | | |  |  |  |  | 0.347 |
| Male | | | 191(36.9) | 138(38.2) | 53(34.0) |  |  |
| Female | | 326(63.1) | | 223(61.8) | 103(66.0) |  |  |
| **RBC** | | 4.48(3.76, 5.30) | | 4.48(3.89, 5.28) | 4.49(3.62, 5.33) | -0.606 | 0.544 |
| **MCV** | | 71.80(64.70,79.70) | | 71.48(64.49,79.40) | 73.35(65.45,80.30) | -1.041 | 0.298 |
| **MCH** | | 21.20(19.00,24.10) | | 21.20(19.00,24.10) | 21.00(18.98,23.97) | 0.216 | 0.829 |
| **RET%** | 1.05(0.60,1.67) | | | 1.01(0.60, 1.64) | 1.07(0.60,1.76) | -0.655 | 0.512 |
| **IRF (%)** | 16.50(11.00,26.40) | | | 16.90(11.60,26.32) | 16.20(10.10,26.85) | -0.716 | 0.474 |
| **LFR (%)** | 82.96(73.10,88.59) | | | 82.30(73.20,88.00) | 83.65(72.70,89.50) | -0.664 | 0.507 |
| **MFR (%)** | | 13.07 (9.30, 16.20) | | 13.10 (9.70, 16.20) | 13.00 (8.98, 16.38) | -0.813 | 0.416 |
| **HFR (%)** | | | 3.10 (1.39, 9.90) | 3.10 (1.40, 9.80) | 3.10 (1.37, 10.43) | -0.067 | 0.947 |
| **Group,n (%)** | | |  |  |  | χ² = 1.153 | 0.283 |
| IDA | | 320 (61.9) | | 218 (60.4) | 102 (65.4) |  |  |
| TT | | 197 (38.1) | | 143 (39.6) | 54 (34.6) |  |  |
| **TT Subtype, n (%)** | |  | |  |  | χ² = 3.238 | 0.356 |
| α-thalassemia deletion | | 105 (53.3) | | 78 (54.5) | 27 (50.0) |  |  |
| β-thalassemia mutation | | 88 (44.7) | | 62 (43.4) | 26 (48.1) |  |  |
| α-thalassemia mutation | | 3 (1.5) | | 2 (1.4) | 1 (1.9) |  |  |
| α+β double thalassemia | | 1 (0.5) | | 1 (0.7) | 0 (0.0) |  |  |

Data are presented as median (Q1, Q3) or n (%).

Abbreviations: RBC, red blood cell; MCV, mean corpuscular volume; MCH, mean corpuscular hemoglobin; RET%, reticulocyte percentage; IRF, immature reticulocyte fraction; LFR, low fluorescence reticulocyte; MFR, medium fluorescence reticulocyte; HFR, high fluorescence reticulocyte; TT, thalassemia trait; IDA, iron deficiency anemia

Figure S1. Variable selection using LASSO regression.

(A) LASSO coefficient profiles of the nine candidate variables. The x-axis represents the log(λ) value, and the y-axis represents the coefficient values. Each colored line represents a variable, with coefficients shrinking toward zero as λ increases.

(B) Ten-fold cross-validation for tuning parameter selection. The x-axis represents log(λ), and the y-axis represents the binomial deviance. The vertical dashed lines indicate λ.min (left) and λ.1se (right). Four variables with non-zero coefficients were selected at λ.1se = 0.078.

Figure S2. CONSORT flow diagram of patient recruitment and dataset partitioning

A total of 705 patients with suspected thalassemia (TT) or iron deficiency anemia (IDA) were initially screened. After eligibility evaluation, 188 patients were excluded for predefined reasons, and 517 eligible subjects were included, consisting of 197 TT patients and 320 IDA patients. The whole cohort was stratified and randomly divided into training set (n=361) and internal validation set (n=156) at a ratio of 7:3.
